# Sanitary Legislation

**Published:** 1857-04

**Authors:** 


### SANITARY LEGISLATION.

The dissolution of Parliament, and the events preceding have stopped the progress of all active sanitary legislation. Still one or two interesting documents have been published bearing on this subject. Mr. Gamgee has addressed a bold and admirable letter to Sir George Grey, on the subject of "*Diseased Animal Food.*" He shows that diseased beasts—some hide-bound with abscesses in various parts of their bodies; others labouring under typhus and various loathsome diseases—are constantly being brought into the London markets, sold, and cut up for human food. He does not flinch at naming such inspectors and officials as are incompetent for their duties; he points out the inefficiency of the laws for preventing the disgusting traffic in diseased animals; shows the impending danger in the possible propagation of the cattle murrain; and suggests that, in London, all private slaughterhouses should be abolished, and be replaced by public ones. If anything could rouse a minister to the exertion of ministerial power, this letter is the very thing to do it. But in England the people do the reforming work; and to them we recommend Mr. Gamgee to submit his appeal. Here it requires the voices of millions to enliven a single "Right Honourable"; on the continent a single voice rules millions. This is the grand political fact for enthusiastic reformers on this side the channel. The nation wills, the minister obeys.

Medical Organisation of the Russian Army. An interesting Report on this subject has been drawn up by Dr. Mouatt and Mr. Wyatt, and presented, through Dr. Andrew Smith, to the Minister of War. It seems that the Russian army, during the Crimean campaign, suffered mainly from intermittent fever. The hygiene of the army was evidently not effective, but was much more thoughtfully considered than our own. Their medical officers were intelligent, but too few in numbers; they were seldom consulted in the selection of sites for camps, and were, on the whole, badly treated. The huts for the men were ill ventilated; some of the hospitals were arranged with care, but were sadly overcrowded; while the dietary system, both for the healthy and sick, appears to have been founded on anything but physiological data. We regret that we are prevented, by a want both of space and of time, from referring at greater length to this document.

The Drainage of the Metropolis question remains under the consideration of the new Commissioners, who are open, we believe, as yet, to the reception of plans. The papers published in this number of our Review, afford a slight indication of the great differences of opinion which prevail regarding this important undertaking; and, as the Commissioners are doubtless overwhelmed with plans, based on views equally conflicting, their decision cannot be expected speedily.

